# A Comparison of Initial Antiretroviral Therapy in the Swiss HIV Cohort Study and the Recommendations of the International AIDS Society-USA

**DOI:** 10.1371/journal.pone.0027903

**Published:** 2011-12-20

**Authors:** Gilles Wandeler, Olivia Keiser, Bernard Hirschel, Huldrych F. Günthard, Enos Bernasconi, Manuel Battegay, Olivier Clerc, Pietro L. Vernazza, Hansjakob Furrer, the Swiss HIV Cohort Study

**Affiliations:** 1 Clinic for Infectious Diseases, Bern University Hospital and University of Bern, Bern, Switzerland; 2 Institute of Social and Preventive Medicine, University of Bern, Bern, Switzerland; 3 University Hospital Geneva, Geneva, Switzerland; 4 Division of Infectious Diseases and Hospital Epidemiology, University Hospital Zurich, University of Zurich, Zurich, Switzerland; 5 Regional Hospital, Lugano, Switzerland; 6 Division of Infectious Diseases and Hospital Epidemiology, University Hospital Basel, Basel, Switzerland; 7 University Hospital Lausanne, Lausanne, Switzerland; 8 Cantonal Hospital, St.Gallen, Switzerland; McGill University AIDS Centre, Canada

## Abstract

**Background:**

In order to facilitate and improve the use of antiretroviral therapy (ART), international recommendations are released and updated regularly. We aimed to study if adherence to the recommendations is associated with better treatment outcomes in the Swiss HIV Cohort Study (SHCS).

**Methods:**

Initial ART regimens prescribed to participants between 1998 and 2007 were classified according to IAS-USA recommendations. Baseline characteristics of patients who received regimens in violation with these recommendations (violation ART) were compared to other patients. Multivariable logistic and linear regression analyses were performed to identify associations between violation ART and (i) virological suppression and (ii) CD4 cell count increase, after one year.

**Results:**

Between 1998 and 2007, 4189 SHCS participants started 241 different ART regimens. A violation ART was started in 5% of patients. Female patients (adjusted odds ratio aOR 1.83, 95%CI 1.28–2.62), those with a high education level (aOR 1.49, 95%CI 1.07–2.06) or a high CD4 count (aOR 1.53, 95%CI 1.02–2.30) were more likely to receive violation ART. The proportion of patients with an undetectable viral load (<400 copies/mL) after one year was significantly lower with violation ART than with recommended regimens (aOR 0.54, 95% CI 0.37–0.80) whereas CD4 count increase after one year of treatment was similar in both groups.

**Conclusions:**

Although more than 240 different initial regimens were prescribed, violations of the IAS-USA recommendations were uncommon. Patients receiving these regimens were less likely to have an undetectable viral load after one year, which strengthens the validity of these recommendations.

## Introduction

After 1996, the use of combined antiretroviral therapy (ART) transformed HIV infection from a progressive, fatal disease to a treatable chronic condition and dramatically reduced its related morbidity and mortality [1], [2], [3]. By analogy to other chronic diseases, there was a need for evidence-based treatment recommendations in order to guide clinicians in their decision-making process thus improving general HIV infection management [4]. Several national and international organisations released recommendations which were updated at short intervals. Although these treatment guidelines provide valuable, evidence or expert opinion based information, they are not always followed by clinicians [5].

In 2001, the Swiss Federal Office of Public Health stopped issuing recommendations [6]. However, the International AIDS Society-USA has been publishing recommendations at least every two years since 1998 [7], [8], [9], [10], [11], [12], [13], [14], [15]. These guidelines were well accepted in Switzerland.

Even though comprehensive treatment recommendations are now widely available, choosing an initial antiretroviral combination often remains a challenging task. In the industrialized world, the availability of more than twenty different antiretroviral drugs has led to highly personalized ART prescriptions, according to patient characteristics and wishes, co-morbidities and resistance testing. This contrasts to the public health approach to ART, as shown by Keiser et al., who compared initial ART regimens prescribed in South Africa and the SHCS [16]. Many more first-line combinations were available in Switzerland compared to South Africa (36 vs. 4), and treatment changes within the first two years were more frequent in Switzerland while virological outcomes were similar in both populations.

CD4 cell count, viral load and socio-demographic characteristics such as age and ethnicity have been shown to be associated with the type of ART prescribed and the probability of being treated with an ART in violation of current guidelines [17], [18], [19].

The main goal of official treatment guidelines is to improve the choice of ART. A publication from a small US cohort showed that patients receiving ART according to the IAS recommendations had clear virological and immunological benefits [19]. Initial treatment efficacy also depends on a wide range of socio-demographic and clinical baseline characteristics. According to a recent systematic review of initial ART studies, higher treatment success rates are seen in “non-white” patients and those with low CD4 cell counts, dual nucleoside backbone and NNRTI or ritonavir-boosted PI based combinations [20].

The aims of this study were to (1) describe first-line ART regimens in the SHCS from 1998 to 2007 and compare them with the corresponding IAS-USA recommendations, (2) describe the socio-demographic and clinical characteristics of the HIV-infected sub-population that did not receive regimens consistent with the IAS-USA recommendations, and (3) compare the efficacy of regimens prescribed according to these recommendations with those in violation with them.

## Methods

### Ethics statement

Local ethical committees of all seven participating study sites (Kantonale Ethikkommission Bern, Ethikkommission beider Basel (EKBB), Ethikkommission des Kantons St. Gallen, Kantonale Ethikkommission Zürich, Comitato etico cantonale del Ticino, Commission d'éthique de la recherche clinique de la faculté de biologie et de medicine de l'université de Lausanne, Comité d'éthique du départment de médicine des hôpitaux universitaires de Genève) have approved the study and written consent has been obtained from all participants.

### Swiss HIV Cohort Study

Set up in 1988, the Swiss HIV Cohort Study (SHCS, www.shcs.ch) is a prospective cohort study with the ongoing enrolment of HIV-positive adults in seven outpatient clinics and their affiliated hospitals and private practice physicians in Switzerland. It is estimated to cover about 45% of the cumulative number of HIV infections declared to the Swiss public health authorities and 69% of all patients living with AIDS in Switzerland [21]. Detailed information on demographics, mode of HIV acquisition, recent risk behavior (for example intravenous drug use or unprotected sex with occasional partners), clinical events and treatment is collected using a standard protocol at registration and then at intervals of 6 months. Details of the study protocol can be found elsewhere [21]. CD4 counts, HIV viral load (Roche Amplicor HIV-1 Monitor Assay) and other laboratory results are obtained every 3 to 6 months. ART is documented in detail at every clinical visit and every change in treatment is registered in the SHCS database. HIV resistance data can be linked with the SHCS database [22]. All services, including antiretroviral therapy and laboratory testing, are covered by compulsory health insurance.

### Study population

Of the 4304 treatment-naïve patients (SHCS download July 2010) who started ART between 01.08.1998 and 31.12.2007, 4189 were included in the analysis. Of the 115 patients that were excluded, 8 received unspecified ART and the remaining 107 patients were women who started treatment because of pregnancy while having a CD4 count above 200 cells/µl. Unlike patients with severe immune deficiency, many of these women started treatment because of the pregnancy and stopped it after having given birth. These women could therefore not be compared with the other patients.

### Classification of ART regimens

Initial ART regimens were compared with the newest IAS recommendations available at treatment start date. Updated recommendations were taken into account from the first day of the month following the publication. ART regimens were classified by two experienced physicians as follows: 1) recommended regimen; 2) alternative regimen; 3) active but not yet recommended regimen; 4) drug combination suggesting primary resistance (in most cases, regimens consisting of more than 3 drugs); 5) prescription within a study protocol; 6) violation of recommendations. Any regimen consisting of one or two active drugs only were considered as violations of the recommendations. In 1998, only PI-based regimens were recommended. In 2000, NNRTI-based regimens were added to the recommended treatments list and triple-NRTI regimens were only accepted as first-line treatments in the 2002 recommendations. We encountered several difficulties in the classification process. There were inconsistencies between the presentations of the different recommendations from one year to the other. For instance, the recommendations elaborated in 2000 did not mention any alternative treatments, and the ones released in 2002 did not specify which combinations of NRTI were to be used or avoided. Importantly, new drugs and combinations are approved all year long but are only included in the subsequent guidelines. To avoid classifying such new, active and safe regimens as violations of recommendations, we created a separate category for them (category 3: active but not yet recommended regimen).

### Statistical analyses

All statistical analyses were performed using Stata 10 (Stata Corp, College Station, USA). Baseline characteristics of patients receiving violation treatments were compared to all other patients (categories 1–5). Crude associations between baseline characteristics and initial regimen type were evaluated using chi-square tests. Predictors of receiving a violation regimen were then assessed in a multivariable logistic regression analysis. Explanatory variables included sex, age category at treatment start (18–30, 31–40, 41–50 or over 50 years old), region of origin (Western Europe, Sub-Saharan Africa or other), risk group according to mode of HIV acquisition (Men who have Sex with Men (MSM), heterosexual, Injection Drug Users (IDU) or other), source of follow-up (SHCS center or other), CD4 count at treatment initiation (<200, 200–350 or >350 cells/µl), hepatitis B antigenemia at baseline (defined as having had at least once a positive HBsAg), chronic hepatitis C virus (HCV) infection (defined as positive HCV serology and HCV RNA), plasma HIV load at baseline (<4, 4–5 or >5 log/ml), education level (low or high education) and start of ART more than three months before being included in the SHCS.

The proportion of patients achieving undetectable viral load after 12 months of ART (closest value between 10 and 18 months), as well as the CD4 increase during this period, were compared between patients receiving violation ART and the others, using multivariable logistic and linear regression analyses respectively. An undetectable viral load was defined as being less than 400 copies/ml. In a sensitivity analysis, a threshold of 50 copies/ml was used. Time to switching of ART was evaluated using a Cox proportional hazards model.

Multiple imputation was used to impute missing values at baseline. Imputations were based on logistic and linear regression models with six dependent variables (region of origin, source of follow-up, CD4 count at start, active hepatitis B infection, log viral load at baseline and education level) and six independent variables (violation of recommendations, sex, treatment initiation period, age at ART start, transmission group and start of ART 3 or more months before entering the SHCS). Analyses were run on each of 25 datasets and results were combined with Rubin's rules. In a sensitivity analysis we also compared these results with a complete case analysis.

## Results

### Baseline characteristics

Baseline socio-demographic and clinical characteristics of the 4,189 HIV-infected patients who started ART between August 1998 and December 2007 are listed in **Table 1**. At the time of initiation of therapy, almost half of the patients had an advanced immune deficiency (CD4 cells below 200/µl) and 20% had already suffered from an AIDS-defining condition.

**Table 1:** Baseline characteristics of patients who initiated ART in the Swiss HIV Cohort Study between 1998 and 2007.

	1998–2000	2000–2002	2002–2004	2004–2006	2006–2007	Total	p-value
Baseline characteristics	N (%)	N (%)	N (%)	N (%)	N (%)	N (%)	
**Sex**							0.17
Male	611 (69.3)	712 (65.8)	529 (66.6)	581 (70.3)	421 (69.6)	**2854 (68.1)**	
Female	271 (30.7)	370 (34.2)	265 (33.9)	245 (29.7)	184 (30.4)	**1335 (31.9)**	
**Age at start**							<0.001
18–30	203 (23.0)	219 (20.2)	189 (23.8)	158 (19.1)	116 (19.2)	**885 (21.1)**	
31–40	421 (47.7)	517 (47.8)	307 (38.7)	304 (36.8)	221 (36.5)	**1770 (42.3)**	
41–50	147 (16.7)	222 (20.5)	187 (23.6)	242 (29.3)	173 (28.6)	**971 (23.2)**	
>50	111 (12.6)	124 (11.5)	111 (14.0)	122 (14.8)	95 (15.7)	**563 (13.4)**	
**Region of origin**							0.01
NW Europe	610 (69.2)	705 (65.2)	516 (65.1)	513 (62.2)	382 (63.1)	**2726 (65.1)**	
Sub-Saharan Africa	100 (11.3)	178 (16.5)	137 (17.3)	134 (16.2)	101 (16.7)	**650 (15.5)**	
Other	172 (19.5)	199 (18.4)	140 (17.7)	178 (21.6)	122 (20.2)	**811 (19.4)**	
**Ethnicity**							0.01
Caucasian	696 (78.9)	808 (74.7)	578 (72.8)	625 (75.7)	430 (71.1)	**3137 (74.9)**	
Other	186 (21.1)	274 (25.3)	216 (27.2)	201 (24.3)	175 (28.9)	**1052 (25.1)**	
**Risk**							<0.001
MSM	298 (33.8)	292 (27.0)	259 (32.6)	318 (38.5)	263 (43.5)	**1430 (34.1)**	
Heterosexual	341 (38.7)	498 (46.0)	394 (49.6)	358 (43.3)	258 (42.6)	**1849 (44.1)**	
IDU	207 (23.5)	246 (22.7)	109 (13.7)	114 (13.8)	54 (8.9)	**730 (17.4)**	
Other	36 (4.1)	46 (4.3)	32 (4.0)	36 (4.4)	30 (5.0)	**180 (4.3)**	
**Source of follow-up**							0.003
SHCS Center	536 (69.5)	694 (70.9)	534 (72.1)	563 (72.3)	425 (72.8)	**2752 (71.4)**	
Other hospital	86 (11.2)	88 (9.0)	45 (6.1)	47 (6.0)	35 (6.0)	**301 (7.8)**	
Private practitioner	149 (19.3)	197 (20.1)	162 (21.9)	169 (21.7)	124 (21.2)	**801 (20.8)**	
**CD4 count at start**							<0.001
<200	362 (47.6)	544 (56.1)	381 (52.3)	371 (47.7)	224 (38.4)	**1882 (49.3)**	
200–349	181 (23.8)	247 (25.5)	219 (30.1)	286 (36.8)	244 (41.9)	**1177 (30.8)**	
> = 350	217 (28.6)	178 (18.4)	128 (17.6)	121 (15.6)	115 (19.7)	**759 (19.9)**	
**AIDS defining condition before start**	196 (22.2)	221 (20.4)	151 (19.0)	165 (20.0)	99 (16.4)	**832 (19.9)**	0.08
**Active hepatitis B infection**	60 (7.1)	72 (6.9)	49 (6.3)	47 (5.9)	30 (5.1)	**258 (6.4)**	0.54
**Active hepatitis C infection**	147 (16.7)	165 (15.3)	117 (14.7)	117 (14.2)	57 (9.4)	**603 (14.4)**	0.002
**Log viral load at start**							<0.001
≤4	176 (23.3)	184 (19.1)	151 (20.5)	174 (22.4)	161 (28.0)	**846 (22.2)**	
4–5	304 (40.3)	347 (36.0)	233 (31.7)	262 (33.7)	206 (35.8)	**1352 (35.5)**	
>5	274 (36.3)	432 (44.9)	351 (47.8)	342 (44.0)	209 (36.3)	**1608 (42.3)**	
**Education**							0.06
no completed school	89 (10.5)	118 (11.3)	84 (10.9)	67 (8.2)	46 (7.7)	**404 (9.9)**	
mandatory school	555 (65.3)	673 (64.5)	475 (61.5)	530 (65.1)	377 (63.2)	**2610 (64.0)**	
higher education	206 (24.2)	252 (24.2)	214 (27.7)	217 (26.7)	174 (29.2)	**1063 (26.1)**	
**ART start ≥3 months before SHCS inclusion**	198 (22.5)	282 (26.1)	221 (27.8)	166 (20.1)	89 (14.7)	**956 (22.8)**	<0.001

### Classification of initial regimens

The classification of initial ART regimens according to the IAS-USA recommendations in force at time of ART start is listed in **Table 2**. Overall, 241 different regimens were prescribed during the study period, the 10 most frequent ones covering two thirds of the patients. Seventy-three percent of patients started one of the recommended first-line treatments and 5.2% of the participants started a regimen in violation with the recommendations. Patients that started treatment between 1998 and 2002 as well as in2007 were more likely to have regimens considered to be in violation with the recommendations. Of the 217 patients who initiated a violation regimen, 53% started a treatment consisting of less than 3 drugs. Fifty-five patients (25%) received a dual NRTI treatment; the most frequently prescribed being the combination of zidovudine and lamivudine (32 patients). Of note, the proportion of dual regimen was highest before 2002. Thirty-four percent of the patients on violation ART were prescribed a regimen containing 3 drugs or more but including an NRTI backbone in violation to IAS-USA recommendations. Only one patient was prescribed a PI-monotherapy during the study period.

**Table 2:** Description of Treatment categories according to the International AIDS Society-USA recommendations.

Period	Number of regimens	Initial treatment categories						
		recommended	alternative	active but not yet recommended	primary resistance	prescription within a study	violation	total
1998–2000	96	483 (55.1)	78 (8.9)	168 (19.2)	65 (7.4)	11 (1.3)	72 (8.2)	**877**
2000–2002	103	762 (70.0)	0 (0)	200 (18.5)	50 (4.6)	10 (0.9)	65 (6.0)	**1087**
2002–2004	64	649 (81.7)	2 (0.3)	104 (13.1)	18 (2.3)	0 (0)	21 (2.6)	**794**
2004–2006	82	668 (80.9)	88 (10.7)	0 (0)	17 (2.1)	31 (3.8)	22 (2.7)	**826**
2006–2007	61	496 (82.0)	57 (9.4)	0 (0)	15 (2.5)	0 (0)	37 (6.1)	**605**
**Total**	**241**	**3058 (73.0)**	**225 (5.4)**	**472 (11.3)**	**165 (3.9)**	**52 (1.2)**	**217 (5.2)**	**4189**

Pearson chi2: p<0.001.

### Predictors of recommendation violations

**Table 3** shows the baseline characteristics of the 4189 patients who started ART during the study period and their association with the use of violation regimens. After adjustment for all covariables, the odds of receiving a treatment combination in violation with the most recent IAS-USA recommendations were higher for women and in highly educated people. Patients who had a CD4 count above 350 cells/µl at ART start were more likely and participants with a viral load between 4 and 5 logs were less likely to receive violation regimens. Those who initiated treatment between 2002 and 2006 had higher odds of being prescribed ART according to the recommendations. Finally, those who started ART more than three months before being included in the SHCS were more often prescribed violation regimens. In the complete case analysis including only the 3363 participants (80%) in whom all variables were measured, there were no major differences in the associations described above (results not shown).

**Table 3:** Predictors for receiving an initial ART regimen in violation with the IAS-USA recommendations (N = 4,189).

Basic characteristics	Treatment		Univariable analysis		Multivariable analysis	
	recommended	Violation	OR (95%CI)	p-value	OR (95% CI)	p-value
**Period**						
1998–2000	810 (91.8)	72 (8.2)	ref.	<0.001	ref.	<0.001
2000–2002	1017 (94.0)	65 (6.0)	0.72 (0.51–1.02)		0.72 (0.50–1.03)	
2002–2004	773 (97.4)	21 (2.6)	0.31 (0.19–0.50)		0.30 (0.18–0.49)	
2004–2006	804 (97.3)	22 (2.7)	0.31 (0.19–0.50)		0.31 (0.19–0.52)	
2006–2007	568 (93.9)	37 (6.1)	0.73 (0.49–1.11)		0.76 (0.49–1.16)	
**Sex**						
Male	2728 (95.6)	126 (4.4)	ref.	0.001	ref.	0.001
Female	1244 (93.2)	91 (6.8)	1.58 (1.20–2.09)		1.83 (1.28–2.62)	
**Age at start**						
18–30	829 (93.7)	56 (6.3)	ref.	0.22	ref.	0.85
31–40	1678 (94.8)	92 (5.2)	0.81 (0.58–1.14)		0.91 (0.63–1.30)	
41–50	924 (95.2)	47 (4.8)	0.75 (0.51–1.12)		1.05 (0.68–1.62)	
>50	541 (96.1)	22 (3.9)	0.60 (0.36–1.00)		0.91 (0.53–1.58)	
**Region of origin**						
NW Europe	2592 (95.1)	134 (4.9)	ref.	0.55	ref.	0.73
Sub-Saharan Africa	612 (94.1)	38 (5.9)	1.20 (0.83–1.74)		1.15 (0.72–1.83)	
Other	766 (94.5)	45 (5.5)	1.14 (0.80–1.61)		1.14 (0.79–1.65)	
**Risk**						
MSM	1360 (95.1)	70 (4.9)	ref.	0.24	ref.	0.11
Heterosexual	1759 (95.1)	90 (4.9)	0.99 (0.72–1.37)		0.73 (0.47–1.13)	
IDU	681 (93.3)	49 (6.7)	1.40 (0.96–2.04)		1.20 (0.77–1.88)	
Other	172 (95.6)	8 (4.4)	0.90 (0.43–1.91)		0.66 (0.29–1.46)	
**Source of follow-up**						
SHCS Center	2622 (95.3)	130 (4.7)	ref	0.20	ref.	0.85
other	1039 (94.3)	63 (5.7)	1.22 (0.90–1.67)		1.03 (0.75–1.42)	
**CD4 count at start**						
<200	1814 (96.4)	68 (3.6)	ref.	0.004	ref.	0.10
200–349	1122 (95.3)	55 (4.7)	1.31 (0.91–1.88)		1.34 (0.91–1.97)	
> = 350	709 (93.4)	50 (6.6)	1.88 (1.29–2.74)		1.53 (1.02–2.30)	
**Active HBV infection**						
No	3593 (94.9)	191(5.1)	ref	0.59	ref.	0.57
Yes	243 (94.2)	15 (5.8)	1.16 (0.68–2.00)		1.18 (0.67–2.06)	
**Log viral load at start**						
< = 4	792 (93.6)	54 (6.4)	ref	0.01	ref.	0.09
4–5	1301 (96.2)	51 (3.8)	0.57 (0.39–0.85)		0.62 (0.40–0.95)	
>5	1544 (96.0)	64 (4.0)	0.61 (0.42–0.88)		0.70 (0.46–1.08)	
**Education**						
no or low education	2873 (95.3)	141 (4.7)	ref.	0.04	ref.	0.02
higher education	996 (93.7)	67 (6.3)	1.37 (1.02–1.85)		1.49 (1.07–2.06)	
**ART ≥3 mo. before inclusion**						
No	3093 (95.7)	140 (4.3)	ref	<0.001	ref.	<0.001
Yes	879 (91.9)	77 (8.1)	1.94 (1.45–2.58)		1.98 (1.45–2.70)	

### Comparison of outcomes one year after treatment initiation

Patients who initiated ART in the SHCS between 1998 and 2007 had a median CD4 count increase of 165 cells/µl (IQR 76-273) within one year, and 85% of them had a viral load below 400 copies/ml one year after treatment start. Patients given violation regimens were less likely to achieve an undetectable viral load (OR: 0.54, 95%CI 0.37–0.80) one year after treatment start (**Table 4**). The initiation of therapy after 2002, a high education level and being treated in a peripheral SHCS site were all associated with having an undetectable viral load after one year of therapy (**online supporting information: Table S1**). This was also true for patients older than 40 years, compared to those under 30. Patients originating from Sub-Saharan Africa were less likely to achieve an undetectable viral load compared to those from Western Europe, as were IDU's compared to MSM. Finally, participants starting ART more than 3 months before enrolment in the SHCS or with a CD4 count above 350 cells/µl were least likely to have an undetectable viral load after one year.

**Table 4:** Virological and immunological outcomes one year after ART start.

Outcome	Univariable analysis		Multivariable analysis	
	OR (95%CI)	p-value	OR (95% CI)	p-value
**Virologically suppressed (<400 copies/mL) 1 year after ART start** **(N = 3,643)**Violation regimen				
No	ref.	<0.001	ref.	0.002
Yes	0.47 (0.33, 0.68)		0.54 (0.37, 0.80)	
**Average CD4 count change (cells/µl)** **1 year after ART start** **(N = 3,171)**Violation regimen				
No	ref.	0.03	ref.	0.11
Yes	−32.9 (−63.3, −25)		−23.9 (−53.2, 5.4)	

Immunological recovery one year after ART start was similar in both treatment groups (**Table 4**). The CD4 cell count increased by 185 cells/µl (95%CI: 179–192) in patients on recommended regimens and by 152 cells/µl (119–185) in those on violation regimens. The strongest predictors of CD4 cell count increase were baseline CD4 count and viral load, calendar time and age at start, as well as transmission risk group (**online supporting information: Table S2**).

Within the first year of treatment, 34% of patients on violation regimens were switched to a second regimen, as compared to 25% in the other groups. The corresponding adjusted hazard ratio in the Cox regression model was 1.66 (95% CI 1.25–2.20; p<0.001).

### Sensitivity analyses

When the threshold of HIV viral load was set at 50 copies/ml instead of 400, the difference in the proportion of patients having a favourable outcome at one year between the two treatment groups was not statistically significant. With this second definition, participants with initial treatment in violation of the recommendations were 25% less likely to reach an undetectable viral load compared to those on recommended regimens, after adjustment for all co-factors (aOR 0.75, 95%CI 0.53–1.06). When analyzed separately, patients on dual-NRTI regimen were also less likely to reach an undetectable viral load (<400copies/mL) after 1 year than patients on recommended regimens (aOR 0.44, 95%CI 0.18–1.08).

## Discussion

Since 1998 and the first version of the IAS-USA recommendations, more than four thousand treatment-naïve HIV-infected patients started ART in the SHCS until the end of 2007, being prescribed one of more than two hundred different regimens. The majority of these participants received initial treatment according to IAS-USA recommendations in force at the time ART was started, as well as according to IAS-USA recommendations on antiretroviral drug resistance testing if primary antiretroviral resistance was shown [23], [24], [25]. Throughout the whole study period, 5% of patients received regimens in violation with these recommendations. In a large cohort of HIV infected US veterans, it was found that between 1998 and 2004, no patient was treated with a regimen that violated protocols and that less than 3% of participants started a regimen with modest activity [18]. The differences between studies comparing and evaluating ART guidelines might be explained by differences in HIV cohorts, but probably to a greater extent by variations in interpretation and use of international recommendations.

The baseline characteristics of the patients receiving violation combinations were of special interest, since it was suspected that these regimens might lead to a higher proportion of viral failures. In our study, these patients were more often female and highly educated. Even though it is unknown why these sub-populations were more likely to receive initial ART in violation of recommendations, physicians should be aware of these associations in order to avoid the prescription of these inferior regimens.. Highly educated patients may also have been more active in the negotiations regarding the choice of the initial treatment regimen with the physician, favouring sometimes regimens known to be of low virological efficacy. In their small study of self-reported initial treatment in a US-cohort of HIV infected women, Cocohoba et al. reported a significant association between the prescription of violation regimens in patients with a higher CD4 count and lower viral load at baseline [19]. Similarly, we found that a CD4 cell count above 350 cell/µl was a predisposing factor for receiving such unfavorable regimens. Previous studies showed a high variability of ART prescription in relation to the year of treatment initiation, violation regimens being used more frequently in the early years of the combination ART era [18], [19]. In the SHCS, patients who started ART in the late 90's were significantly more likely to receive a violation regimen than those who started after 2000, possibly because of the physician's lack of experience with combination ART and international treatment recommendations. It also seems that the aim of suppressing viral load to levels below 50 copies/ml, was less prominent in the early years of combination ART, as shown by the significant proportion of dual ART regimens reported for the first period in this study.

In the SHCS, patients treated according to the IAS-USA recommendations between 1998 and 2007 were significantly more likely to achieve an undetectable viral load (<400 cp/mL) after one year, compared with those on a violation regimen. Importantly, patients on violation regimens were more likely to change their treatment to a different regimen during the first six months of ART. As a consequence, changes from a violation regimen to a recommended one might have had a positive impact on clinical outcomes in this sub-group of patients. This might have decreased the difference in virological outcome between patients on violation ART and those on recommended regimens. When the definition of an undetectable viral load was restricted to less than 50 copies/ml, the association between treatment group and virological outcome was not statistically significant. Even though this second definition is widely used in the clinical settings, it may not be appropriate in this context, as some patients might experience single episodes of low-level viremia, so-called blips, which often do not have any clinical consequences. Notably, IDU's and patients from Sub-Saharan Africa were less likely to have an undetectable viral load one year after treatment start compared to the other risk groups, even though they were not prescribed violation regimens more often. These associations have both been described in previous studies and might be explained by lower treatment adherence, as described in the SHCS by Glass et al. [26], [27]

Another relevant finding of this study relates to the important role of the SHCS in the daily clinical management of HIV infected patients in Switzerland. Participants who started ART more than 3 months before being included in the SHCS were more often prescribed regimens in violation with the recommendations and were less likely to have an undetectable viral load after one year. Although this seems to support the management of HIV infected patients by physicians experienced in the field of HIV medicine, this finding could also be explained by differences in access to care or other socio-economic factors.Finally, immunological response to ART in patients on violation regimens showed a less favorable trend compared to CD4 recovery in patients on recommended initial ART, although the difference was not statistically significant in the multivariable model. As expected, younger patients and those with a low CD4 count at baseline had a better immunologic recovery than the others.

There are several limitations to this study. First, it has to be noted that our classification of the different initial ART regimens in the SHCS was based on expert judgement and not on pre-specified objective criteria. Second, detailed description of antiretroviral combinations is not available in every set of recommendations thus allowing for a wider range of treatments accepted in the different treatment categories depending on the year of release. Third, our statistical analyses rely on observational data, which leads to different types of bias. For example, data on patient history is only collected twice a year, and is therefore subject to recall bias. CD4 counts and viral load values were not always obtained exactly 12 months after ART initiation, the ones measured closest to this time period had to be used. Finally, the logistic regression models cannot be adjusted for every single existing confounder, which means that residual confounding has to be taken into account when interpreting the results. Finally, given the large amount of ART-related issues discussed in the IAS-USA treatment recommendations, we could not consider all determinants of ART prescription for our analyses. As a consequence, even though possible adverse events and pill count, for instance, should be considered when choosing the appropriate initial ART regimen, we decided to limit our analyses to the description of individual predictors for receiving violation regimens and the evaluation of the virological and immunological response to the different ART regimens.

In summary, our results show that in the SHCS, 5% of patients received initial regimens in violation with the IAS-USA recommendations. These patients were less likely to achieve viral load suppression after one year. Our results suggest that, in the context of constant increase in the number of therapeutic options and knowledge on specific drug-related side-effects and interactions, the release of updated treatment recommendations as well as the promotion of their use are important to guarantee the best possible care of HIV infected patients.

## Supporting Information

Table S1 **Univariable and multivariable regression models estimating the proportion of patients having an undetectable viral load (<400 copies/mL) one year after ART start.**
(DOCX)

Table S2 **Univariable and multivariable regression models estimating the average change in CD4 cell count one year after initial ART start.**
(DOCX)
