# Supplementary material for: A Comparison of Initial Antiretroviral Therapy in the Swiss HIV Cohort Study and the Recommendations of the International AIDS Society-USA
Source: PLoS One. 2011 Dec 20;6(12):e27903. doi: 10.1371/journal.pone.0027903 (PMC3243684; doi:10.1371/journal.pone.0027903)
Supplement: Table S1 — Univariable and multivariable regression models estimating the proportion of patients having an undetectable viral load (<400 copies/mL) one year after ART start. (DOCX) [file pone.0027903.s001.docx]

| **Online supporting information:Table S1. Univariable and multivariable regression models estimating the proportion of patients having an undetectable viral load (<400 copies/mL) one year after ART start** | | | | | |
| --- | --- | --- | --- | --- | --- |
| **Basic characteristics** | **Number** | **Univariable analysis** | | **Multivariable analysis on imputed data (N=3643)** | |
|  | **(%)** | OR (95%CI) | p-value | OR (95% CI) | p-value |
| **Violation of guidelines** | (N=3643) |  |  |  |  |
| No | 2993 (86%) | ref. | <0.001 | ref. | 0.002 |
| Yes | 123 (75%) | 0.47 (0.33, 0.68) |  | 0.54 (0.37, 0.80) |  |
|  |  |  |  |  |  |
| **Period** | (N=3643) |  |  |  |  |
| 1998-2000 | 552 (79%) | ref. | <0.001 | ref. | <0.001 |
| 2000-2002 | 705 (80%) | 1.11 (0.87, 1.41) |  | 1.06 (0.82, 1.37) |  |
| 2002-2004 | 632 (88%) | 1.96 (1.47, 2.62) |  | 1.82 (1.34, 2.46) |  |
| 2004-2006 | 704 (91%) | 2.68 (1.98, 3.63) |  | 2.35 (1.71, 3.24) |  |
| 2006-2007 | 523 (92%) | 2.94 (2.08, 4.16) |  | 2.48 (1.73, 3.56) |  |
|  |  |  |  |  |  |
| **Sex** | (N=3643) |  |  |  |  |
| Male | 2164 (87%) | ref. | 0.002 | ref. | 0.84 |
| Female | 952 (83%) | 0.74 (0.61, 0.90) |  | 0.98 (0.77, 1.24) |  |
|  |  |  |  |  |  |
| **Age at start** | (N=3643) |  |  |  |  |
| 18-30 | 596 (81%) | ref. | <0.001 | ref. | 0.01 |
| 31-40 | 1260 (84%) | 1.24 (0.99, 1.56) |  | 1.16 (0.91, 1.49) |  |
| 41-50 | 796 (90%) | 2.24 (1.68, 2.98) |  | 1.72 (1.26, 2.35) |  |
| >50 | 464 (90%) | 2.16 (1.54, 3.03) |  | 1.37 (0.94, 1.99) |  |
|  |  |  |  |  |  |
| **Region of origin** | (N=3641) |  |  |  |  |
| NW Europe | 2066 (86%) | ref. | 0.02 | ref. | 0.11 |
| Sub-Saharan Africa | 462 (82%) | 0.71 (0.55, 0.90) |  | 0.71 (0.52, 0.98) |  |
| Other | 586 (85%) | 0.90 (0.71, 1.14) |  | 0.93 (0.72, 1.21) |  |
|  |  |  |  |  |  |
| **Risk** | (N=3643) |  |  |  |  |
| MSM | 1150 (90%) | ref. | <0.001 | ref. | <0.001 |
| Heterosexual | 1419 (86%) | 0.70 (0.56, 0.89) |  | 0.92 (0.69, 1.23) |  |
| IDU | 409 (74%) | 0.31 (0.25, 0.41) |  | 0.40 (0.30, 0.54) |  |
| Other | 138 (85%) | 0.65 (0.41, 1.04) |  | 0.81 (0.49, 1.33) |  |
|  |  |  |  |  |  |
| **Source of follow-up** | (N=3367) |  |  |  |  |
| SHCS Center | 2075 (85%) | ref. | 0.03 | ref. | 0.02 |
| other | 828 (88%) | 1.28 (1.02, 1.61) |  | 1.34 (1.05, 1.71) |  |
|  |  |  |  |  |  |
| **CD4 count at start** | (N=3421) |  |  |  |  |
| <200 | 1478 (88%) | ref. | <0.001 | ref. | <0.001 |
| 200-349 | 949 (89%) | 1.08 (0.85, 1.37) |  | 0.93 (0.73, 1.20) |  |
| >=350 | 531 (79%) | 0.50 (0.39, 0.63) |  | 0.48 (0.37, 0.62) |  |

| **Table S1. *(continued)*** **Univariate and multivariable regression models estimating the proportion of patients having an undetectable viral load one year after ART start** | | | | | |
| --- | --- | --- | --- | --- | --- |
| **Basic characteristics** | **Number** | **Univariable analysis** | | **Multivariable analysis on imputed data (N=3643)** | |
|  | **(%)** | OR (95% CI) | p-value | OR (95% CI) | p-value |
| **Active hepatitis B infection** | (N=3538) |  |  |  |  |
| No | 2845 (86%) | ref. | 0.03 | ref. | 0.17 |
| Yes | 191 (81%) | 0.68 (0.49, 0.96) |  | 0.78 (0.54, 1.11) |  |
|  |  |  |  |  |  |
| **Log viral load at baseline** | (N=3418) |  |  |  |  |
| <= 4 | 656 (85%) | ref. | 0.24 | ref. | 0.49 |
| 4-5 | 1047 (87%) | 1.23 (0.94, 1.59) |  | 1.10 (0.83, 1.45) |  |
| >5 | 1263 (87%) | 1.21 (0.94, 1.55) |  | 0.95 (0.72, 1.26) |  |
|  |  |  |  |  |  |
| **Education** | (N=3553) |  |  |  |  |
| no or low education | 2192 (84%) | ref. | <0.001 | ref. | 0.001 |
| higher education | 850 (91%) | 1.92 (1.50-2.45) |  | 1.57 (1.20, 2.04) |  |
|  |  |  |  |  |  |
| **ART start ≥3 months before SHCS inclusion** | (N=3643) |  |  |  |  |
| No | 2546 (87%) | ref. | <0.001 | ref. | <0.001 |
| Yes | 570 (80%) | 0.60 (0.49, 0.74) |  | 0.59 (0.47, 0.75) |  |
