# Supplementary material for: A Comparison of Initial Antiretroviral Therapy in the Swiss HIV Cohort Study and the Recommendations of the International AIDS Society-USA
Source: PLoS One. 2011 Dec 20;6(12):e27903. doi: 10.1371/journal.pone.0027903 (PMC3243684; doi:10.1371/journal.pone.0027903)
Supplement: Table S2 — Univariable and multivariable regression models estimating the average change in CD4 cell count one year after initial ART start. (DOCX) [file pone.0027903.s002.docx]

| **Online supporting information: Table S2. Univariable and multivariable regression models estimating the average change in CD4 cell count one year after initial ART start** | | | | | | | | | |
| --- | --- | --- | --- | --- | --- | --- | --- | --- | --- |
| **Basic characteristics** | **Univariable analysis** | | | | | **Multivariable analysis on imputed data (N=3171)** | | | |
|  | Mean (95% CI) | | p-value | | Mean (95% CI) | | | p-value | |
| **Violation of guidelines** | (N=3171) | |  | |  | |  |  | |
| No | ref. | | 0.03 | | ref. | | | 0.11 | |
| Yes | -32.9 (-63.3, -25) | |  | | -23.9 (-53.2, 5.4) | | |  | |
|  |  |  |  | |  | |  |  | |
| **Period** | (N=3171) | |  | |  | |  |  | |
| 1998-2000 | ref. | | <0.001 | | ref. | | | <0.001 | |
| 2000-2002 | -3.8 (-23.0, 15.3) | |  | | -11.3 (-29.8, 7.2) | | |  | |
| 2002-2004 | 20.2 (0.3, 40.1) | |  | | 9.5 (-10-0, 29.0) | | |  | |
| 2004-2006 | 32.5 (12.9, 52.1) | |  | | 22.0 (2.8, 41.2) | | |  | |
| 2006-2007 | 46.7 (25.7, 67.7) | |  | | 38.5 (18.0, 59.0) | | |  | |
|  |  |  |  | |  | |  |  | |
| **Sex** | (N=3171) | |  | |  | |  |  | |
| Male | ref. | | 0.91 | | ref. | | | 0.02 | |
| Female | -0.7 (-14.1, 12.7) | |  | | 18.9 (3.6, 34.3) | | |  | |
|  |  |  |  | |  | |  |  | |
| **Age at start** | (N=3171) | |  | |  | |  |  | |
| 18-30 | ref. | | 0.21 | | ref. | | | <0.001 | |
| 31-40 | -10.3 (-27.3, 6.7) | |  | | -13.8 (-30.5, 2.8) | | |  | |
| 41-50 | -13.5 (-32.4, 5.33) | |  | | -23.6 (-42.7, -4.5) | | |  | |
| >50 | -23.1 (-44.8, -1.4) | |  | | -45.3 (-67.6,-22.9) | | |  | |
|  |  |  |  | |  | |  |  | |
| **Region of origin** | (N=3170) | |  | |  | |  |  | |
| NW Europe | ref. | | 0.03 | | ref. | | | 0.04 | |
| Sub-Saharan Africa | -10.8 (-28.2, 6.7) | |  | | -16.8 (--37.1, 3.4) | | |  | |
| Other | 17.5 (1.2, 33.8) | |  | | 11.4 (-4.8, 27.5) | | |  | |
|  |  |  |  | |  | |  |  | |
| **Risk** | (N=3171) | |  | |  | |  |  | |
| MSM | ref. | | <0.001 | | ref. | | | <0.001 | |
| Heterosexual | -26.7 (-40.7, -12.8) | |  | | -26.3 (-43.0, -9.6) | | |  | |
| IDU | -80.1 (-99.2, -61.1) | |  | | -74.3 (-94.3, -54.3) | | |  | |
| Other | -13.5 (-44.5, 17.5) | |  | | -9.9 (-40.8, 21.0) | | |  | |
|  |  |  |  | |  | |  |  | |
| **Source of follow-up** | (N=2943) | |  | |  | |  |  | |
| SHCS Center | ref. | | 0.57 | | ref. | | | 0.43 | |
| other | -4.2 (-18.8, 10.4) | |  | | -5.7 (-19.9, 8.4) | | |  | |
|  |  |  |  | |  | |  |  | |
| **CD4 count at start** | (N=3171) | |  | |  | |  |  | |
| <200 | ref. | | <0.001 | | ref. | | | <0.001 | |
| 200-349 | 10.4 (-4.0, 24.7) | |  | | 16.8 (2.6, -30.9) | | |  | |
| >=350 | -31.55 (-48.1, -15.0) | |  | | -16.5 (-33.0, -0.1) | | |  | |
|  |  |  | |  | |  |  | |  |

| **Table S2. *(continued)* Univariable and multivariable regression models estimating the average change in CD4 cell count one year after initial ART start** | | | | | | |
| --- | --- | --- | --- | --- | --- | --- |
| **Basic characteristics** | **Univariable analysis** | | | **Multivariable analysis on imputed data (N=3171)** | | |
|  | Mean (95% CI) | | p-value | Mean (95% CI) | | p-value |
| **Active hepatitis B infection** | (N=3080) | |  |  |  |  |
| No | ref. | | <0.001 | ref. | | 0.001 |
| Yes | -53.8 (-79.7, -27.9) | |  | -43.0 (-67.9, -18.1) | |  |
|  |  |  |  |  |  |  |
| **Log viral load at baseline** | (N=3136) | |  |  |  |  |
| <= 4 | ref. | | <0.001 | ref. | | <0.001 |
| 4-5 | 44.9 (28.5, 61.3) | |  | 41.5 (25.0, 57.9) | |  |
| >5 | 104.3 (88.4, 120.1) | |  | 103.5 (87.3, 119.8) | |  |
|  |  |  |  |  |  |  |
| **Education** | (N=3091) | |  |  |  |  |
| no or low education | ref. | | 0.05 | ref. | | 0.83 |
| higher education | 14.4 (0.1, 28.7) | |  | 1.6 (-12.8, 15.9) | |  |
|  |  |  |  |  |  |  |
| **ART start ≥3 months before SHCS inclusion** | (N=3171) | |  |  |  |  |
| No | ref. | | 0.02 | ref. | | 0.003 |
| Yes | -19.3 (-35.9, -2.7) | |  | -25.0 (-41.3, -8.7) | |  |
